# Phenolic Lipids Affect the Activity and Conformation of Acetylcholinesterase from *Electrophorus electricus* (Electric eel)

**DOI:** 10.3390/nu6051823

**Published:** 2014-04-30

**Authors:** Maria Stasiuk, Alicja Janiszewska, Arkadiusz Kozubek

**Affiliations:** Department of Lipids and Liposomes, Faculty of Biotechnology, University of Wroclaw, Joliot-Curie 14a, Wroclaw 50-383, Poland; E-Mails: ala_j@vp.pl (A.J.); kozubek@ibmb.uni.wroc.pl (A.K.)

**Keywords:** phenolic lipids, resorcinolic lipids, acetylcholinesterase, Trp fluorescence

## Abstract

Phenolic lipids were isolated from rye grains, cashew nutshell liquid (CNSL) from *Anacardium occidentale*, and fruit bodies of *Merrulius tremellosus*, and their effects on the electric eel acetylcholinesterase activity and conformation were studied. The observed effect distinctly depended on the chemical structure of the phenolic lipids that were available for interaction with the enzyme. All of the tested compounds reduced the activity of acetylcholinesterase. The degree of inhibition varied, showing a correlation with changes in the conformation of the enzyme tested by the intrinsic fluorescence of the Trp residues of the protein.

## 1. Introduction

The phenolic lipids are a heterogeneous group of natural lipids, including simple phenols, polyphenols and their derivatives. A certain sub-population of single-ring phenolic lipids are specifically found in certain plants and these contain a catechol, resorcinol or hydroquinone moiety alk(en)ylated by a multicarbon chain [1]. Phenolic lipids were reported to have antiparasitic, anticancer, antifungal, antimicrobial and antioxidant effects. Their various biological activities suggest their involvement in regulating the metabolism at the cellular and organism levels [2].

**Figure 1:**
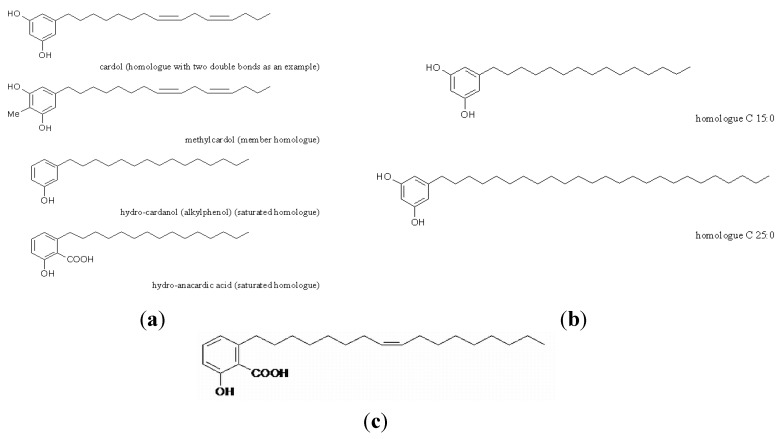
The structures of the studied compounds. (**a**) Resorcinolic and alkylphenolic lipids from *Anacardium occidentale*. (**b**) Resorcinolic lipids from rye grain (C15:0 and C25:0 as an example). (**c**) Merulinic acid from *Merulius tremellosus*.

Anacardic acids, cardols, cardanols and methylcardols (Figure 1a) are the main non-isoprenoid phenolic lipid components of cashew nutshell liquid (CNSL). Anacardic acid has been used to generate other potentially bioactive compounds (salicylate macrolactones) [3]. Resorcinolic lipids (ARs, Figure 1b) are present in many higher plants, but also in bacteria, fungi, algae and mosses [1]. They are most common in the bran of cereal grains [4]. When present in the diet, alkylresorcinols have been found to be absorbed not only by rats and pigs [5], but also by humans [6,7,8], and they are transported via the lymphatic system and carried in association with erythrocyte membranes and lipoproteins in the blood [8,9,10]. Merulinic acid (Figure 1c) from *Merulius tremellosus* seems to be one of the most biologically active phenolic lipids [2,11].

Acetylcholine (ACh) is a universal cell molecule found in all plants and animals and it plays a role in neuronal and non-neuronal signaling [12]. It is also widely accepted to have a role in non-neuronal tissue, including tumors [13,14], in hematocytopoietic disorders [15], and in regulation of the immune function [16]. After each nerve transmission, ACh is inactivated through enzymatic degradation mediated by cholinesterases. It was hypothesized that the cognitive loss associated with Alzheimer’s disease (AD) is related to a reduction in the acetylcholine levels and a central cholinergic deficit. Thus, increasing the amount of ACh using acetylcholinesterase inhibitors might enhance cognitive function in AD patients [17,18].

Acetylcholinesterase (AChE) is a conservative enzyme encoded by a single gene. It is a product of alternative splicing and therefore, can be bound to the membrane via a glycosyl-phosphatidylinositol anchor positioned in the exoplasmic leaflet of the membrane (erythrocyte AChE) or PRIMA peptide (synaptic AChE) or soluble in water (e.g., “read through” AChE). Each form has identical catalytic properties regardless of the place of appearance [19]. The well-known AChE inhibitors, such as Tacrin, Donepezil, Rivastigmin, Galantamine, Huperzine, Physo- and Eptastigmine, inhibit enzyme activity through interaction with the protein part of the enzyme [20]. In previous studies [11,21], we observed a decrease in the activity of GPI-anchored erythrocyte AChE in the presence of phenolic lipids. Later studies on GPI-deprived AChE did not allow a precise determination as to whether the inhibition occurs as a result of phenolic lipid interactions with the protein part of the enzyme or through modulation of the membrane properties [22]. The aim of this study was to determine if phenolic lipids might interact directly with the protein part of AChE and if this affects the activity of this enzyme.

## 2. Materials and Methods

### 2.1. Chemicals

Electric eel AChE (>1000 U/mg protein), acetylthiocholine iodide and DTNB (5,5-dithiobis(2-nitro)-benzoic acid) were purchased from Sigma-Aldrich, Poznan, Poland. AChE solution was received at a concentration of 1.5 mg/mL and was diluted 1500 times (for the estimation of AChE activity) or 75 times (for Trp fluorescence tests).

Homologues of the phenolic lipids, resorcinolic lipids and merulinic acid were isolated chromatographically according to procedures described previously (respectively in [23,24,25]). The purity of the tested compounds was assessed by HPLC and was above 98%. All of the lipids were used as methanolic solutions.

The remaining chemicals were of the best available purity and obtained from CHEMPUR (Piekary Slaskie, Poland).

### 2.2. Estimation of AChE Activity

AChE activity was assayed using the method described by Ellman [26]. Briefly, acetylthiocholine is used as the substrate, and the product, thiocholine, as the indicator of enzymatic activity after its reaction with DTNB to form a yellow anion, 5-thio-2-nitrobenzoic acid. First, 20 μL of AChE and DTNB with Na_2_CO_3_ (to final concentrations of 320 and 450 μM, respectively) were added to 3 mL of 0.1 M Na_2_HPO_4_/NaH_2_PO_4_ (pH 8.0). Microliter amounts (less than 50 μL) of the investigated compounds (concentration range from 8 to 94 μM) were then added. After 5 min of pre-incubation, acetylcholine iodide was added to a final concentration of 0.476 μM.

The progress of acetylthiocholine iodide hydrolysis was recorded spectrophotometrically with a Shimadzu UV-2401 PC (Shimadzu Co., Kyoto, Japan), UV–VIS Recording Spectrophotometer at 37 °C. Changes in the absorbance of the samples at 412 nm were recorded continuously for 10 min. The rate of the reaction (V) was calculated as follows:

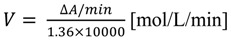
(1)
where ΔA is the increase in absorbance of the sample over 1 min at 37 °C.

The enzyme activity modulated by the studied compounds was calculated as a percentage of the control reaction. The influence of the solvent and lipids was considered in the control and in blank samples.

### 2.3. Estimation of the Effect of Phenolic Lipids on Intrinsic Fluorescence of Trp Residues of AChE

Alterations in the microenvironment of the Trp of the peptide upon interactions with the tested compounds were monitored as described previously [27]. Small amounts (less than 50 μL) of phenolic lipids in methanolic solution (in the range of 20–213 μM) were added to 2 mL of the reaction mixture containing 0.1 M Na_2_HPO_4_/NaH_2_PO_4_ (pH 8.0) and 25 μL of AChE with continuous stirring, and maintained at 24 °C. After 10 min of equilibration, Trp fluorescence spectra were measured with a Varian Cary Eclipse spectrofluorometer (Varian, Mulgrave, Victoria, Australia). The tryptophan residues were excited at 295 nm and emission spectra were recorded from 300 to 400 nm. Spectra were corrected for the contribution of light scattering in the presence of the tested lipids.

## 3. Results

**Figure 2:**
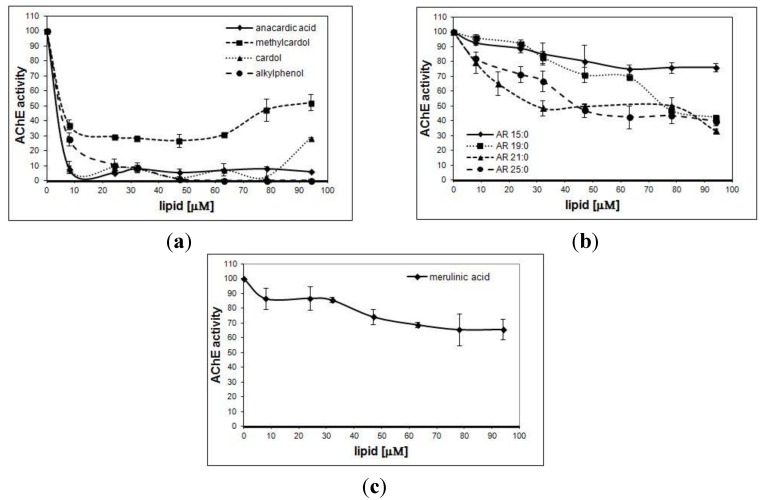
Inhibitory effect of phenolic lipids on acetylcholine (AChE) from E. electricus. (**a**) Phenolic resorcinolic and alkylphenolic lipids from *A. occidentale*. (**b**) Resorcinolic lipids from rye grain. (**c**) Merulinic acid from *M. tremellosus*. Data are given as means ± SD of three individual determinations, each performed in triplicate.

Alkylphenolic (cardanol and anacardic acid) and resorcinolic lipids (cardol and methylcardol) from cashew nutshell liquid (CNSL) (Figure 1a), alkylresorcinols from rye grain (Figure 1b) and merulinic acid from *Merulius tremellosus* (Figure 1c) were tested for their *in vitro* inhibitory activity on AChE from *Electrophorus electricus*. The results are expressed as graphs (Figure 2a–c respectively). A decreased activity of AChE was observed in the presence of all of the tested phenolic lipids in the experimental media. The most effective decrease was observed after the addition of phenolic lipids from *A. occidentale*: anacardic acid, cardol and alkylphenol inhibited the enzyme activity almost completely. Resorcinolic lipids from rye bran demonstrated AChE activity inhibition that was related to the length of the alkyl chain present in the molecule. The increase in the number of carbon atoms in the chain caused a greater inhibition of the enzyme, but it never exceeded 70%. Merulinic acid caused AChE inhibition by max. 40%.

The most effective inhibitors of AChE are the CNSL-derived lipids, which exhibit IC_50_ (the inhibitor concentration causing 50% inhibition) of similar values. The IC_50_ values of rye bran lipids are ten times higher (Table 1).

**Figure 3:**
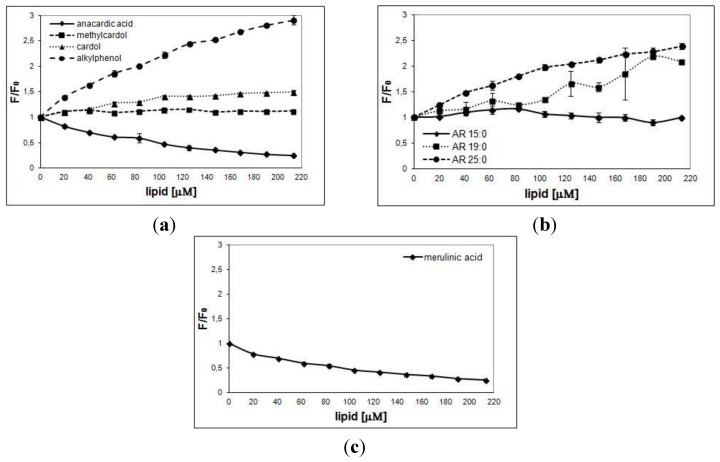
Interaction of *E. electricus* AChE with phenolic lipids. The graphs show the ratio of the fluorescence emission intensity at 310 nm of AChE *versus* lipid concentration. (**a**) Resorcinolic and alkylphenolic lipids from *A. occidentale*. (**b**) Resorcinolic lipids from rye grain. (**c**) Merulinic acid from *M. tremellosus*. F_0_ and F are the fluorescence intensity in the absence and in the presence of phenolic lipids, respectively. Data are given as means ± SD of three individual determinations, each performed in triplicate.

Changes in the Trp emission of the investigated proteins in the presence of phenolic lipids depended on the chemical structures of compound applied. The more evident conformational modification was observed after treatment with alkylphenol (the value of F/F_0_ is three times greater, Figure 3a), AR 25:0 (the value of F/F_0_ is 2.5 times greater) and AR 19:0 (the value of F/F_0_ is 2 times higher, Figure 3b). The value of F/F_0_ decreases by about 80% after the addition of anacardic and merulinic acids (Figure 3a,c respectively). The change in fluorescence of Trp residues are smaller for the other lipids tested. No significant statistical differences were observed in fluorescence maxima position during our experiments.

**Table 1:** AChE inhibition (IC50 values, μM) of phenolic lipids.

Studied Compound	IC_50_ ± SD [μM]
Cardol	3.5 ± 0.2
Methylcardol	5 ± 0.41
Alkylphenol	4 ± 0.24
Anacardic acid	3 ± 0.24
AR 15:0	>94
AR 19:0	77 ± 1.25
AR 21:0	44 ± 0.47
AR 25:0	44 ± 4.24
Merulinic acid	>94

## 4. Discussion

The characteristics parameters of Trp fluorescence are sensitive to changes in the microenvironment of this amino acid side chain, such as those caused by alterations in peptide conformation during interactions with ligands. It is known that the fluorescence intensity of Trp residues is strongly affected by the characteristics of its environment, such as dielectric constant. Lowering the polarity of the environment (and dielectric constant at the same time) causes a decrease in the fluorescence intensity of Trp residues [28,29]. The decrease in fluorescence intensity may be due both to changes in protein conformation (and changes in Trp residues exposure to an aqueous solution) as well as the direct quenching of fluorescence by the added ligands [30].

The tested compounds that do not have a polar charged part of the molecule (ARs from rye bran, alkylphenol and cardol from CNSL) cause an increase in the value of F/F_0_. By contrast, the lipids with a polar part of the carboxyl group (anacardic and merulinic acids) cause a decrease in the value of F/F_0_. The presence of methyl residues at the 1,3-dihydroxybenzene ring (methylcardol) does not alter the conformation of the protein.

The results suggest that in the case of molecules with a side chain of the same length (lipids from CNSL), the effect depends on the chemical structure of the polar parts of the molecule. On the other hand, in the case of ARs from rye bran, which have the same polar head, the effect is dependent on the length of the side chain. The change in the value of F/F_0_ becomes more significant with the increase in the number of carbon atoms in the side chain of the lipid molecule. This means that the mechanism of interactions of phenolic lipids with AChE is more complex, with the enzyme probably affecting both polar and non-polar parts of the lipid molecules.

There is a correlation between the observed changes in the value of AChE inhibition and value of F/F_0_ after addition of phenolic lipids to the reaction medium. Lipids that cause a change in the conformation of the enzyme also inhibit its activity. The exceptions are methylcardol and AR 15:0, which do not cause changes in the fluorescence of Trp residues even though they inhibit the activity of the enzyme in the ranges of 50%–70% and 20%, respectively. Earlier studies have indicated several major domains within the protein: a catalytic active site composed of two sub-sites, the aromatic gorge in which the catalytic active site lies, and a peripheral anionic site, distinct from the catalytic active site [31]. The active site is composed of two sub-sites: the esteratic sub-site which contains the catalytic triad, and the anionic sub-site that accommodates the positive quaternary pole of acetylcholine. The esteratic sub-site contains the catalytic machinery of the enzyme: a catalytic triad of Ser200, His440, and Glu327. The anionic sub-site is defined by Trp84, Phe330, and Phe331. Its role is to orient the charged part of the substrate that enters the active centre. This role is the main function of the Trp84 residue [31]. The peripheral anionic site contains Trp286, and is able to bind to many different types of ligands, and by doing so affects the conformation of the active centre. This site exhibits flexibility which accommodates many distinct ligands, and also implies their conformational mobility [32]. No shift on the band maximum was observed, which may suggests that the mechanism of the Trp residues quenching is complex (simultaneous quenching by direct interaction and possible modification of the conformation) [30].

## 5. Conclusions

It has been suggested that phenolic lipids alter the activity of GPI-anchored erythrocyte AChE by changing the physicochemical properties of the phospholipid bilayer. Our experiments use AChE from *E. electricus* as a model and indicated that the phenolic lipids may also interact directly with the protein part of this enzyme. Our results suggest that the effect of phenolic lipids on GPI-anchored erythrocyte AChE is probably more complicated and dependent on a direct interaction of lipid molecules with the protein part of the enzyme and on changes in the physicochemical properties of the phospholipid bilayer.

## References

[1] Kozubek A., Tyman J.H.P. (1999). Resorcinolic lipids, the natural non-isoprenoic amphiphiles and their biological activity. Chem. Rev..

[2] Stasiuk M., Kozubek A. (2010). Biological activity of phenolic lipids. Cell. Mol. Life Sci..

[3] Logrado L.P.L., dos Santos M.L., Silveira D., Romeiro L.A.S., Moraes M.O., Cavalcanti B.C., Costa-Lotufo L.V., do Ó Pessoa C., dos Santos M.L. (2005). Synthesis and biological evaluation of new salicylate macrolactones from anacardic acids. J. Braz. Chem. Soc..

[4] Ross A.B., Shepherd M.J., Schupphaus M., Sinclair V., Alfaro B., Kamal Eldin A., Aman P. (2003). Alkylresorcinols in cereals and cereal products. J. Agric. Food Chem..

[5] Ross A.B., Shepherd M.J., Bach Knudsen K.E., Glitso L.V., Bowey E., Phillips J., Rowland I., Guo Z.-X., Massy D.J., Aman P. (2003). Absorption of dietary alkylresorcinols in ileal cannulated pigs and rats. Br. J. Nutr..

[6] Linko A.M., Parikka K., Wahala K., Adlercreutz H. (2002). Gas chromatographic-mass spectrometric method for the determination of alkylresorcinols in human plasma. Anal. Biochem..

[7] Linko A.M., Juntunen K.S., Mykkänen H.M., Adlercreutz H. (2005). Whole-grain rye bread consumption by women correlates with plasma alkylresorcinols and increases their concentration compared with low-fiber wheat bread. J. Nutr..

[8] Ross A.B., Kamal-Eldin A., Lundin E.A., Zhang J.X., Hallmans G., Aman P. (2003). Cereal alkylresorcinols are absorbed by humans. J. Nutr..

[9] Linko A.M., Aldercreutz H. (2005). Whole-grain rye and wheat alkylresorcinols are incorporated into human erythrocyte membranes. Br. J. Nutr..

[10] Linko A.M., Landberg R., Tikkanen M.J., Adlercreutz H., Penalvo J.L. (2007). Alkylresircinols from whole-grain wheat and rye are transported in human plasma lipoproteins. J. Nutr..

[11] Stasiuk M., Jaromin A., Kozubek A. (2004). The effect of merulinic acid on biomembranes. Biochim. Biophys. Acta.

[12] Wessler I., Kilbinger H., Bittinger F., Kirkpatrick C.J. (2001). The biological role of non-neuronal acetylcholine in plants and humans. Jpn. J. Pharmacol..

[13] Paleari L., Grozio A., Cesario A., Russo P. (2008). The cholinergic system and cancer. Semin. Cancer Biol..

[14] Silman J., Sussman L. (2005). Acetylcholinesterase: “Classical” and “nonclassical” functions and pharmacology. Curr. Opin. Pharmacol..

[15] Battisti V., Schetinger M.R.C., Maders L.D.K., Santos K.F., Bagatini M.D., Correa M.C., Spanevello R.M., do Carmo Araújo M., Morsch V.M. (2009). Changes in acetylcholinesterase (AchE) activity in lymphocytes and whole blood in acute lymphoblastic leukemia patients. Clin. Chim. Acta.

[16] Razani-Boroujerdi S., Behl M., Hahn F., Pena J.C., Hutt J., Sopori M.L. (2008). Role of muscarinic receptors in the regulation of immune and inflammatory responses. J. Neuroimmunol..

[17] Benzi G., Moretti A. (1998). Is there a rationale for the use of acetylcholinesterase inhibitors in the therapy of Alzheimer’s disease?. Eur. J. Pharmacol..

[18] Mega M.S. (2000). The cholinergic deficit in Alzheimer’s disease: Impact on cognition, behavior and function. Int. J. Neuropsychopharmacol..

[19] Valle A.M., Radic Z., Rana B.K., Mahboubi V., Wessel J., Shih P.B., Rao F., Connor D.T.O., Taylor P. (2011). Naturally occurring variations in the human cholinesterase genes: Heritability and association with cardiovascular and metabolic traits. J. Pharmacol. Exp. Ther..

[20] Sugimoto H., Yamanishi Y., Iimura Y., Kawakami Y. (2000). Donepezil hydrochloride (E2020) and other acetylcholinesterase inhibitors. Curr. Med. Chem..

[21] Stasiuk M., Kozubek A. (2008). Membrane perturbing properties of natural phenolic and resorcinolic lipids. FEBS Lett..

[22] Stasiuk M., Kleta M., Kozubek A. (2011). Dual effect of free and liposomal forms of phenolic lipids on the activity of GPI-anchor-deprived acetylcholinesterase from erythrocytes. Food Chem..

[23] Przeworska E., Gubernator J., Kozubek A. (2001). Formation of liposomes by resorcinolic lipids, single chain phenolic amphiphiles from *Anacardium occidentale* L.. Biochim. Biophys. Acta.

[24] Kozubek A. (1985). Isolation of 5-*n*-alkyl-, 5-*n*-alkenyl- and 5-*n*-alkadienyl-homologs of alk(en)ylresorcinols from rye grains. Acta Aliment. Pol..

[25] Giannetti B.M., Steglich W., Quack W., Anke T., Oberwinkler F. (1978). Antibiotika aus basidiomyceten: VI. Merulinsauren A, B und C, neue antibiotika aus *Merulius tremellosus* Fr. und *Phlebia radiata* Fr.. Z. für Naturforschung.

[26] Ellman G.L., Courtney D., Andres D., Featherstone R.M. (1961). A new rapid colorimetric determination of acetylcholinesterase activity. Biochem. Pharmacol..

[27] Zhao H., Sood R., Jutila A., Bose S., Fimland G., Nissen-Meyer J., Kinnunen P.K.J. (2006). Interaction of the antimicrobial peptide pheromone Plantaricin A with model membranes: Implications for a novel mechanism of action. Biochim. Biophys. Acta.

[28] Liu S., Shibata A., Ueno S., Xu F., Baba Y., Jiang D., Li Y. (2006). Investigation of interaction of Leu-enkephalin with lipid membranes. Colloids Surf. B.

[29] Loura L.M.S., Almeida R.F.M., Coutinho A., Prieto M. (2003). Interaction of peptides with binary phospholipid membranes: Application of fluorescence methodologies. Chem. Phys. Lipids.

[30] Terlecki G., Czapinska E., Rogozik K., Lisowski M., Gutowicz J. (2006). Investigation of the interaction of pig muscle lactate dehydrogenase with acidic phospholipids at low pH. Biochim. Biophys. Acta.

[31] Sussman J.L., Harel M., Frolow F., Oefner C., Goldman A., Toker L., Silman I. (1991). Atomic structure of acetylcholinesterase from Torpedo californica: A prototypic acetylcholine-binding protein. Science.

[32] Ordentlich A., Barak D., Kronman C., Flashner Y., Leitner M., Segall Y., Ariel N., Cohen S., Velan B., Shafferman A. (1993). Dissection of the human acetylcholinesterase active center determinants of substrate specificity. Identification of residues constituting the anionic site, the hydrophobic site, and the acyl pocket. J. Biol. Chem..

